# A multi-similarity spectral clustering method for community detection in dynamic networks

**DOI:** 10.1038/srep31454

**Published:** 2016-08-16

**Authors:** Xuanmei Qin, Weidi Dai, Pengfei Jiao, Wenjun Wang, Ning Yuan

**Affiliations:** 1 School of Computer Software, Tianjin University, Tianjin, 300350, China; 2 School of Computer Science and Technology, Tianjin University, Tianjin, 300350, China; 3 Department of Basic Courses, Academy of Military Transportation, PLA, Tianjin 300161, China

## Abstract

Community structure is one of the fundamental characteristics of complex networks. Many methods have been proposed for community detection. However, most of these methods are designed for static networks and are not suitable for dynamic networks that evolve over time. Recently, the evolutionary clustering framework was proposed for clustering dynamic data, and it can also be used for community detection in dynamic networks. In this paper, a multi-similarity spectral (MSSC) method is proposed as an improvement to the former evolutionary clustering method. To detect the community structure in dynamic networks, our method considers the different similarity metrics of networks. First, multiple similarity matrices are constructed for each snapshot of dynamic networks. Then, a dynamic co-training algorithm is proposed by bootstrapping the clustering of different similarity measures. Compared with a number of baseline models, the experimental results show that the proposed MSSC method has better performance on some widely used synthetic and real-world datasets with ground-truth community structure that change over time.

Complex networks have been studied in many domains, such as genomic networks, social networks, communication networks and co-author networks^1^. The community structure has revealed important structure in these complex networks^2^,^3^,^4^,^5^,^6^. A great deal of research has been devoted to detecting communities in complex networks, such as graph partitioning^7^,^8^, hierarchical clustering^9^, modularity optimization^10^, spectral clustering^11^,^12^, label propagation, game theory and information diffusion^13^, a detailed review is available in the literature^14^. However, most existing methods are designed for static networks, and not suitable for real-world data networks with dynamic characteristics. For example, the interactions among users in the blogosphere or circles of friends are not stationary because some interactions disappear, and some new ones appear each day.

Recently, some methods have been proposed to find community structures and their temporal evolution in dynamic networks. An intuitive idea is to divide the network into discrete time steps and to use static methods to the snapshot networks^15^,^16^,^17^,^18^,^19^,^20^,^21^,^22^. The so-called two-stages methods, analyse the community extraction and the community evolution in two separated stages. In other words, the communities are extracted at a given snapshot while ignoring the changing trends among and within communities of the dynamic networks. These two-stage methods are extremely noise-sensitive and produce unstable clustering results. For example, nodes or links disappear or emerge in the subsequent snapshot, which is impossible to detect using the two-stage methods. A better choice is to consider multiple time steps as a whole and the evolutionary clustering algorithm is proposed^23^, which can detect communities of the current snapshot by joining with the community structure of the previous snapshot.

In fact, evolutionary clustering algorithm enables one to detect current communities using community structures from the previous steps by introducing an item called the temporal smoothness. The general framework for evolutionary clustering was first formulated by Chakrabarti *et al*.^23^. In this framework, they proposed heuristic solutions to evolutionary hierarchical clustering and k-means clustering. The framework FacetNet, which was proposed by Lin *et al*.^24^, relies on non-negative matrix factorization. A density-based clustering method, which was proposed by Kim and Han^25^, and uses a cost embedding technique and optimal modularity, can efficiently find temporally smoothed local clusters of high quality.

The existing evolutionary clustering methods that are most similar to MSSC are the PCQ (preserving cluster quality) and PCM (preserving cluster membership) methods^26^. PCQ and PCM are two proposed frameworks that incorporate the temporal smoothness in spectral clustering. In both frameworks, a cost function is defined as the sum of the traditional cluster quality cost and the temporal smoothness item. Our method follows the evolutionary clustering strategy, but with one major difference. The intuitive goal of spectral clustering is to detect latent communities in networks such that the points are similar in the same community and different in different communities. There are several similarity measurements to evaluate the similarities between two vertices. A common approach is to encode prior knowledge about objects using a kernel, such as the linear kernel, Gaussian kernel and Fisher kernel. A large proportion of existing spectral clustering algorithms use only one similarity measurement. However, there is a problem in that the clustering results based on different similarity matrices may be notably different^11^,^27^. Here, we introduce a multi-similarity method to the evolutionary spectral clustering algorithm, which simultaneously considers multiple similarity matrices.

Inspired by Abhishek Kumar *et al*.^28^, we propose a multi-similarity spectral clustering (MSSC) method and a dynamic co-training algorithm for community detection in dynamic networks. The proposed method preserves the evolutionary information of community structure by combining the current data and historic partitions. The idea of co-training was originally proposed in semi-supervised learning for bootstrapping procedures where two hypotheses are trained in different views^29^. The cotraining idea assumes that the two views are conditionally independent and sufficient, i.e., each view can conditionally independently give the classifiers and be sufficient for classification on its own. Then the classification is restricted in one view to be consistent with those in other views. Co-training has been used to classify web pages using the text on the page as one view and the anchor text of hyperlinks on other pages that point to the page as the other views^30^. In another words, the text in a hyperlink on one page can provide information about the page to which it links. Similarity to semi-supervised learning, the clustering, which is based on different similarity measures, is obtained using information from one another by co-training in the proposed dynamic co-training approach. This process is repeated in a pre-defined number of iterations.

Moreover, the problem of how to determine the weight of the temporal penalty to the historic partitions, which reflects the user preferences on the historic information, remains. In many cases, this parameter depends on the users’ subjective preference^26^, which is undesirable. We propose an adaptive model to dynamically tune the temporal smoothness parameter.

In summary, we introduce multiple similarity measures in the evolutionary spectral clustering method. We propose a dynamic co-training method, which accommodates multiple similarity measures and regularizes current communities according to the temporal smoothness of historic ones. Then, an adaptive approach is presented to learn the change in weight of the temporal penalty over time. Based on these ideas, a multi-similarity evolutionary spectral clustering method is presented to discover communities in dynamic networks using the evolutionary clustering^23^ and dynamic co-training method. The performance of the proposed MSSC method is demonstrated on some widely used synthetic and real-world datasets with ground-truths.

## Results

To quantitatively compare our algorithm and others, we compare the values of the normalized mutual information (NMI)^31^ and the sum of squares for error (SSE)^32^ for various networks from the literature. The NMI is a well known entropy measure in information theory, which measures the similarity of two clusters (in this paper, between the community structures 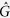 obtained using our method and *G* obtained from the ground truth). Assume that the *i*–*th* row of 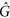 indicates the community membership of the *i*–*th* node (i.e., if the *i*th node belongs to the k-th community, then 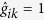 and 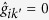 for *k* ≠ *k*′). NMI can be defined as 
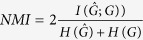
, which is the normalization of mutual information 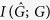 by the average of two entropies 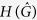 and *H*(*G*). The NMI value is a quantity between 0 and 1, a higher NMI indicates higher consistency, and *NMI* = 1 corresponds to being identical. SSE can be defined as 
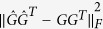
, which measures the distance between the community structure represented by 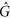 and that represented by *G*. A smaller SSE, indicates a smaller difference between the prediction values and the factual values.

We compare the accuracy against three previously published spectral clustering algorithms for detecting communities in dynamic networks: the preserving cluster quality method (PCQ)^26^, the preserving cluster membership method (PCM)^26^ and the traditional two-stage method. PCQ and PCM are two proposed frameworks that incorporate temporal smoothness in spectral clustering. In both frameworks, a cost function is defined as the sum of the traditional cluster quality cost and a temporal smoothness one. Although these two frameworks have similar expressions for the cost function, the temporal smoothness cost in PCQ is expressed as how well the current partition clusters historic data, which makes the clusters depend on both current data and historic data, whereas the temporal cost in PCM is expressed as the difference between the current partition and the historic partition, which prevents the clusters from dramatically deviating from the recent history. The traditional two-stage method divides the network into discrete time steps and performs static spectral clustering^11^ at each time step. Each approach is repeated for 10 times, and the average result and variance are presented. The parameter for PCQ and PCM is *α* = 0.9. We begin by inferring communities in three synthetic datasets with known embedded communities. Next, we study two real-world datasets, where communities are identified by human domain experts. For concreteness and simplicity, we restrict ourselves in this paper to the case of two similarity measures. The proposed method can be extended for more than two similarity matrices. We choose to use the Gaussian kernel and linear kernel as the similarity measures among different data points. Then, the similarity matrices are 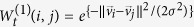 and 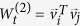, where 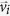 and 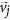 represent the *m*-dimensional feature vectors and *i* ≠ *j*. In our experiments, 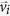 is a column vector of the adjacency matrix *A* at snapshot *t*, which is represented by *A*_*t*_. In other words, 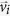 is an *n*-dimensional feature vector. The *σ* is taken equal to the median of the pair-wise Euclidean distances between the data points.

### Synthetic Datasets

#### GN-benchmark network #1

**Figure 1:**
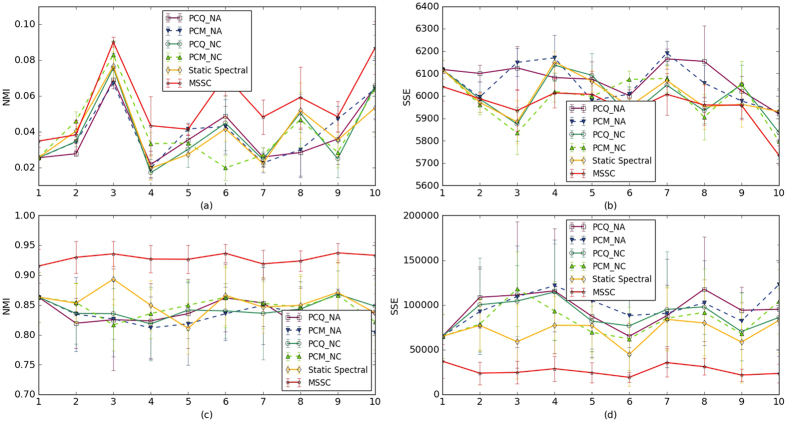
The performance of different methods in synthetic networks. (**a**,**b**) Normalized mutual information and the sum of the squared errors of different methods at 10 snapshots in synthetic networks, where the parameter *z* is 5, the average degree of each node is 16 and at each snapshot, 3 nodes change their cluster membership. (**c**,**d**) Performance for a single contraction event with 1000 nodes over 10 snapshots; the nodes have a mean degree of 15, a maximum degree of 50, and a mixing parameter value of *μ* = 0, which controls the overlapping among communities. Notice that the x-axes show the snapshots.

The first dataset is generated according to the description by Newman *et al*.^33^. This dataset contains 128 nodes, which are divided into 4 communities, each of which has 32 nodes. We generate data for 10 consecutive snapshots. In each snapshot from 2 to 10, the dynamics are introduced as follows: from each community we randomly select certain members to leave their original community and randomly join the other three communities. Pairs of nodes are randomly linked with a higher probability *p*_*in*_ for within-community edges and a lower probability *p*_*out*_ for between-community edges. Aloughth *p*_*out*_ is freely varied, the value of *p*_*in*_ is selected to maintain the expected degree of each vertex as a constant. When the average degree for the nodes is fixed, parameter *z*, which represents the mean number of edges from a node to the nodes in other communities, is sufficient to describe the data. With the increase in *z*, the community structure becomes indistinct. We consider three values (4, 5 and 6) for *z*; the average degree of each node is 16 and 20 at each snapshot. We randomly select 1, 3 and 6 nodes to change their cluster membership. The performance is significantly improved, as shown in Table 1, where *Cn* is the number of changed nodes. In general, our method has a higher NMI and a smaller SSE in most situations except when *z* = 4 (the average degree is 16) and *z* = 5 (the average degree is 20), where the NC-based PCM outperforms the MSSC method. Because space is limited, the values of NMI and SSE are the average values from snapshot 1 to 10. In Fig. 1, we intuitively show the performance under the conditions that parameter *z* is 5, the average degree of each node is 16 and at each snapshot, 3 nodes change their cluster membership. Figure 1(a) shows that the MSSC method always outperforms the baselines, which indicates that our method has a better accuracy. In addition, Fig. 1(b) shows that the MSSC method has a lower error in cluster membership with respect to the ground truth from a general view. In both figures, our method significantly improves the accuracy and reduces the error compared with PCQ, PCM and static spectral clustering.

#### GN-benchmark network #2

To compare the effectiveness as the number of communities varies, we use the second dataset with two types of data sets, which were generated by Francesco Folino and Clara Pizzuti^34^: SYN-FIX with a fixed number of communities and SYN-VAR with a variable number of communities. For SYN-FIX, the data generating method is identical to the GN-benchmark network #1. The network consists of 128 nodes, which are divided into four communities of 32 nodes. Every node has an average degree of 16 and shares *z* links with other nodes of the network. Then, 3 nodes are randomly selected from each community and randomly assigned to the other three communities. For SYN-VAR, the generating method for SYN-FIX is modified to introduce the forming and dissolving of communities and the attaching and detaching of nodes. The initial network contains 256 nodes, which are divided into 4 communities of 64 nodes. Then, 10 consecutive networks are generated by randomly choosing 8 nodes from each community, and a new community is generated with these 32 nodes. This process is performed for 5 timestamps before the nodes return to the original communities. Every node has an average degree of 16 and shares *z* links with the other nodes of the network. A new community is created once at each timestamp between 2 ≤ *t* ≤ 5. Therefore, the numbers of communities between 1 ≤ *t* ≤ 10 are 4, 5, 6, 7, 8, 8, 7, 6, 5, and 4. At each snapshot, 16 nodes are randomly deleted, and 16 new nodes are added to the network for 2 ≤ *t* ≤ 10. Table 2 shows the accuracy and error of the community membership that are obtained by the four algorithms for SYN-FIX and SYN-VAR with *z* = 3 and *z* = 5. Table 2 shows that the MSSC method can handle dynamic networks well when the number of community varies, and when *z* = 3, the community structure is easy to detect because there is less noise. Hence, although MSSC does not perform well in NMI, it has a lower error for SYN-FIX.

#### Synthetic dataset #3

The third synthetic dataset is used to study the MSSC method in dynamic networks, where the number of nodes changes. Greene *et al*.^31^ developed a set of benchmarks based on the embedding of events in synthetic graphs. Five dynamic networks are generated without overlapping communities for five different event types: birth and death, expansion, contraction, merging and splitting, and switch. A single birth event occurs when a new dynamic community appears, and a single death event occurs when an old dynamic community dissolutions. A single mergeing event occurs if two distinct dynamic communities observed at snapshot *t* − 1 match to a single step community at snapshot *t* and a single splitting event occurs if a single dynamic community at snapshot *t* − 1 is matched to two distinct step communities at snapshot *t*. The expansion of a dynamic community occurs when its corresponding step community at snapshot *t* is significantly larger than the previous one and the contraction of a dynamic community occurs when its corresponding step community at snapshot *t* is significantly smaller than the previous one. The switch event occurs when the nodes move among the communities. The performance of a small example dynamic graph produced by the generator is shown in Fig. 1(c,d), which involves 1000 nodes, 17 embedded dynamic communities and a single contraction event. To evaluate methods, we constructed five different synthetic networks for five different event types, which covered 1000 nodes over 10 snapshots. In each of the five synthetic datasets, 20% of node memberships were randomly permuted at each snapshot to simulate the natural movement of users among communities over time. The snapshot graphs share a number of parameters: the nodes have a mean degree of 15, a maximum degree of 50, and a mixing parameter value of *μ* = 0, which controls the overlap between communities. The number of communities were constrained to have sizes in the range of [20, 100]. In each of the five synthetic datasets, the node memberships were randomly permuted at each step to simulate the natural movement of users among communities over time. Table 3 shows the performance of five different methods in different events. We also find that the standard deviation for MSSC is smaller, which implies that the clustering results are more stable.

### Real-World Datasets

#### NEC Blog Dataset

**Figure 2:**
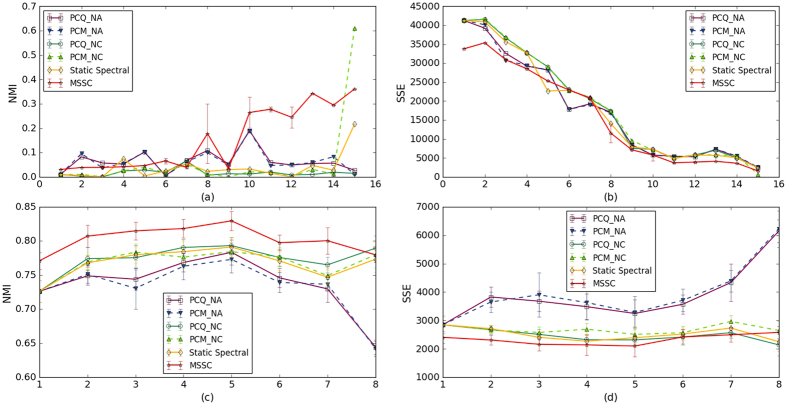
The performance for real-world dataset. (**a**,**b**) This NEC blog dataset contains 407 blogs crawled during 15 consecutive months, which begin from July 2005, where each month is a snapshot. (**c**,**d**) The network of e-mail contacts at the department of computer science at KIT is an ever-changing network during 48 consecutive months, where the snapshot is six months.

The blog data were collected by an NEC in-house blog crawler. Given seeds of manually picked highly ranked blogs, the crawler discovered blogs that were densely connected with the seeds, which resulted in an expanded set of blogs that communicated with each other. The NEC blog dataset has been used in several previous studies on dynamic networks^24^,^26^,^35^. The dataset contains 148, 681 entry-to-entry links among 407 blogs crawled during 15 months, which start from July 2005. First, we construct an adjacency matrix, where the nodes correspond to blogs, and the edges are interlinks among the blogs (obtained by aggregating all entry-to-entry links). In the blog network, the number of nodes changes in different snapshots. The blogs roughly form 2 main clusters, the larger cluster consists of blogs with technology focuses and the smaller cluster contains blogs with non-technology focuses (e.g., politics, international issues, digital libraries). Therefore, in the following studies, we set the number of clusters to be 2. Figure 2 shows the performance. Because the edges are sparse, we take 4 weeks as a snapshot and aggregate all edges in every month into an affinity matrix for that snapshot. Figure 2(a) shows that although MSSC does not perform as well as NA-based PCQ and PCM in the first few snapshots, MSSC begins to outperform NA-based PCQ and PCM as time progresses. In addition, MSSC retains a lower variance than NA-based PCQ and PCM. This result suggests that the benefits of MSSC accumulate more over time than those of NA-based PCQ and PCM. Furthermore, Fig. 2(b) shows that MSSC has lower errors although it does not outperform the baselines in NMI at few snapshots.

#### KIT E-mail Dataset

Furthermore, we consider a large number of snapshots of the e-mail communication network in the Department of Informatics at KIT^36^. The network of e-mail contacts at the department of computer science at KIT is an ever-changing graph during 48 consecutive months from September 2006 to August 2010. The vertices represent members, and the edges correspond to the e-mail contacts weighted by the number of e-mails sent between two individuals. Because the edges are sparse, we construct the adjacency matrix among 231 active members. In the E-mail network, the clusters are different departments of computer science at KIT. The number of clusters is 14, 23, 25, 26, and 27, for the snapshots of 1, 2, 3, 4, and 6 months, respectively, because the smaller divided intervals correspond to more data points that are treated as isolated points. Therefore, when we take one month as a snapshot, the number of clusters is the smallest. Because of limited space, we show the NMI scores and SSE values for the 8 snapshots situation (each snapshot is six months) in Fig. 2(c,d). We observe that MSSC outperforms the baseline methods. To study the effect of considering historic information, Table 4 takes 1, 2, 3, 4, and 6 months as a snapshot. We observe that the more snapshots, correspond to more know historic information and smaller error. Therefore the SSE is smallest when the dynamic networks are considered as 48 snapshots.

## Discussion

In this paper, to find a highly efficient spectral clustering method for community detection in dynamic networks, we propose an MSSC method by considering different measures together. We first construct multiple similarity matrices for each snapshot of dynamic networks and present a dynamic co-training method that bootstrapping the clustering of different similarity measures using information from one another. Furthermore, the proposed dynamic co-training method, which considers the evolution between two neighbouring snapshots can preserve the historic information of community structure. Finally, we use a simple but effective method to adaptively estimate the temporal smoothing parameter in the objective.

We have evaluated our MSSC method on both synthetic and real-world networks with ground-truths, and compared it with three state-of-the-art spectral clustering methods. The experimental results show that the method effectively detects communities in dynamic networks for most analysed data sets with various network and community size.

In all of our experiments, we observe that the major improvement in performance is obtained in the first iteration. The performance varies around that value in subsequent iterations. Therefore, in this paper, we show the results after the first iteration. In general, the algorithm does not converge, which is also the case with the semi-supervised co-training algorithm^28^.

However, the number of clusters or communities must be pre-designed in each snapshot. Determining the number of clusters is an important and difficult research problem in the field of model selection. There is currently no good resolution method for this problem. Some previously suggested approaches to this problem are cross-validation^37^, minimum description length methods that use two-part or universal codes^38^, and maximization of a marginal likelihood^39^. Our algorithms can use any of these methods to automatically select the number of cluster *k* because our algorithm still uses the fundamental spectral clustering algorithm. Additionally, as a spectral clustering method, MSSC must construct an adjacency matrix and calculate the eigen-decomposition of the corresponding Laplacian matrix. Both steps are computationally expensive. For a data set of *n* data points, these two steps have complexities of *O*(*n*^2^) and *O*(*n*^3^), which are unbearable burdens for large-scale applications^40^. There are some options to accelerate the spectral clustering algorithm, such as landmark-based spectral clustering (LSC), which selects 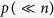 representative data points as the landmarks and represents the remaining data points as the linear combinations of these landmarks^41^,^42^. Liu *et al*.^43^ introduced a sequential reduction algorithm based on the observation that some data points quickly converge to their true embedding, so that an early stop strategy will speed up the decomposition. Yan, Huang, and Jordan^44^ also provided a general framework for fast approximate spectral clustering.

## Methods

### Traditional spectral clustering

In this section, we review the traditional spectral clustering approach^11^. The basic idea of spectral clustering is to cluster based on the spectrum of a Laplacian matrix. Given a set of data points {*x*_1_, *x*_2_, …, *x*_*n*_}, the intuitive goal of clustering is to find a reasonable method to divide the data points into several groups, with greater similarity in each group and dissimilarity among the groups. From the view of graph theory, the data can be represented as a similarity-based graph *G* = (*V, E*) with vertex set *V* and edge set *E*. Each vertex *v*_*i*_ in this graph represents a data point *x*_*i*_, and the edge between vertices *v*_*i*_ and *v*_*j*_ is weighted by similarity *W*_*ij*_. For any given similarity matrix *W*, we can construct the unnormalized Laplacian matrix by *L* = *D* − *W* and the normalized Laplacian matrix by 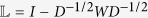, where the degree matrix *D* is defined as a diagonal matrix with elements 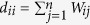. The adjacency matrix is a square matrix *A*, such that its element *A*_*ij*_ is one when there is an edge from vertex *v*_*i*_ to vertex *v*_*j*_ and is zero when there is no edge. Two common variants of spectral clustering are average association and normalized cut^45^. The two partition criteria that maximize the association with the group and minimize the disassociation among groups are identical (the proof is provided in the literature^45^). Unfortunately, each variant is associated with an NP-hard problem. The relaxed problems can be written as^11^,^26^,^45^


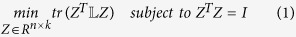


In our algorithm, we will use the normalized cut as the partition criteria. The optimal solution to this problem is to set *Z* to be the eigenvectors that correspond to the k smallest eigenvalues of 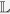. Then, all data points are projected to the eigen-space and the k-means algorithm is applied to the projected points to obtain the clusters. The focus of our work is the definition of the similarity matrix in the spectral clustering algorithm, i.e. computing the relaxed eigenvectors *Z*s with different similarity measurements.

### Different similarity measures

In spectral clustering, a similarity matrix should be constructed to quantify the similarity among the data points. The performance of the spectral clustering algorithm heavily depends on the choice of similarity measures^46^. There are several constructions to transform a given set of data points into their similarities. A common approach in machine learning is to encode prior knowledge about the data vertices using a kernel^27^. The linear kernel which is given by the inner products between implicit representations of data points, is the simplest kernel function. Assume that the *i*th node in *V* can be represented by an *m*-dimensional feature vector 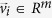, and the distance between the *i*th and *j*th nodes in *V* is 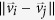, which is the Euclidean distance. The linear kernel can be used as a type of similarity measure, i.e., similarity matrix *W* can be solved by 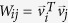. The Gaussian kernel function is one of the most common similarity measures for spectral clustering^11^, which can be written as 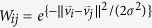, where the standard deviation of the kernel *σ* is equal to the median of the pair-wise Euclidean distances between the data points.

There are also some specific kernels for the similarity matrix. Fischer and Buhmann^47^ proposed a path-based similarity measure based on a connectedness criterion. Chang *et al*.^48^ proposed a robust path-based similarity measure based on the M-estimator to develop the robust path-based spectral clustering method.

Different similarity measures may reveal similarity between data points from different perspectives. For example, the Gaussian kernel function is based on Euclidean distances between the data points, whereas the linear kernel function is based on the inner products of the implicit representations of data points. Most studies of spectral clustering are based on one type of similarity measure, and notably few works consider multiple similarity measures. Therefore, we propose a method to consider multiple similarity measures in spectral clustering. In other words, our goal is to find a spectral clustering method based on multiple similarity matrice.

### Multi-similarity spectral clustering

**Figure 3:**
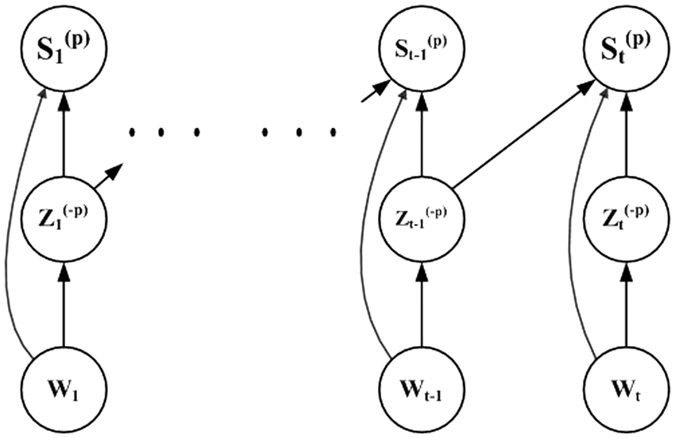
The graphical illustration of the dynamic co-training method. 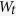
 represents the similarity matrix at snapshot *t* 
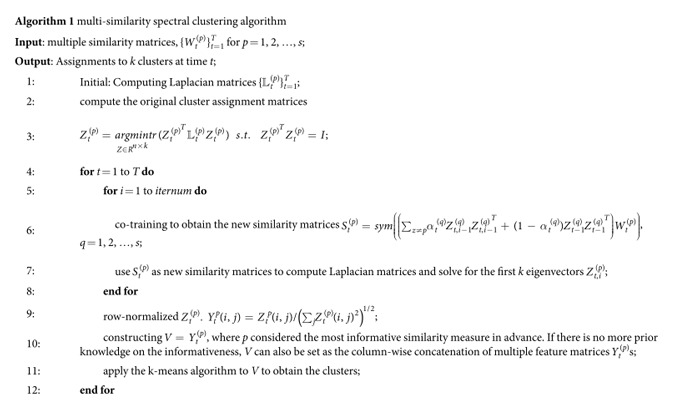
 represents the new similarity matrix after the dynamic co-training. 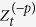 denotes the discriminative eigenvector in the Laplacian matrix obtained from the 1, 2, 3 … *s*th except for the *p*th similarity measures.

First, we introduce basic ideas on multi-similarity spectral clustering in the dynamic networks. We assume that the clustering from one similarity measurement should be consistent with the clustering from the other similarity measurements, and we bootstrapping the clustering of different similarities using information from one another by a dynamic co-training. The dynamic co-training method based on the idea of evolutionary clustering can preserve historic information of community structure. After a new similarity matrix is obtained by the dynamic co-training, we follow the standard procedures in traditional spectral clustering and obtain the clustering result. Figure 3 graphically illustrate the dynamic co-training process.

Specifically, we first compute the similarity matrices with different similarity measures at snapshot *t*, and the *p*th similarity matrix is denoted by 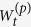. Following most spectral clustering algorithms, a solution to the problem of minimizing the normalized cut is the relaxed cluster assignment matrix 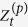 whose columns are the eigenvectors associated with the first *k* eigenvalues of the normalized Laplacian matrix 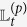. Then all data points are projected to the eigen-space, and the clustering result is obtained usin the k-means algorithm. For a Laplacian matrix with exactly *k* connected components, its first *k* eigenvectors are are the cluster assignment vectors, i.e., these *k* eigenvectors only contain discriminative information among different clusters, while ignoring the details in the clusters^11^. However, if the Laplacian matrix is fully connected, the eigenvectors are no longer the cluster assignment vectors, but they contain discriminative information that can be used for clustering. From the co-training, we can use the eigenvectors from one similarity matrix to update the other one. The updated similarity matrix on the *p*th similarity measure at snapshot *t* can be defined as


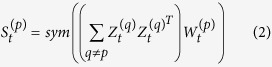



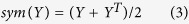


where 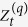 denotes the discriminative eigenvector in the Laplacian matrix from the *q*th similarity measure, *p, q* = 1, 2, 3, … *s* and *p* ≠ *q*. Equation (3) is the symmetrization operator to ensure that the projection of similarity matrix 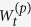 onto the eigenvectors is a symmetric matrix. Then, we use 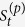 as the new similarity matrix to compute the Laplacians and solve for the first *k* eigenvectors to obtain a new cluster assignment matrix 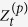. After the co-training procedure is repeated for a pre-selected number of iterations, matrix 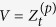 is constructed, where *p* is considered the most informative similarity measure in advance. Alternatively, if there is no prior knowledge of the similarity informativeness, matrix *V* can be set to be the column-wise concatenation of all 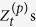. For example, we generate two cluster assignment matrices 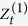 and 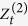, which are combined to form 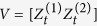. Finally, the clusters are obtained using the k-means algorithm on *V*.

As descibed, we can solve the problem to accommodate multiple similarities. A further consideration is to follow the evolutionary clustering strategy to preserve the historic information of the community structure based on the co-training method. A general framework for evolutionary clustering was proposed by a linear combination of two costs^26^:


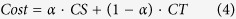


where *CS* measures the snapshot quality of the current clustering result with respect to the current data features, *CT* measures the goodness-of-fit of the current clustering result with respect to either historic data features or historic clustering results.

Here, we assume that the clusters at any snapshot should mainly depend on the current data and should not dramatically shift to the next snapshot. Then, a better approximation to the inner product of the feature matrix and its transposition is define as


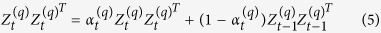


where 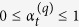, and 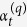 is the temporal penalty parameter that controls the weight on the current information and historic information. Notice that 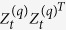 is determined by both current eigenvectors and historic eigenvectors, so the updated similarity 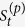 defined in Equation (2), which considers the history, produces stable and consistent clusters. With the increase in 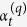, more weight is placed on the current information, and less weight is placed on the historic information. Algorithm 1 describes the MSSC algorithm in detail.


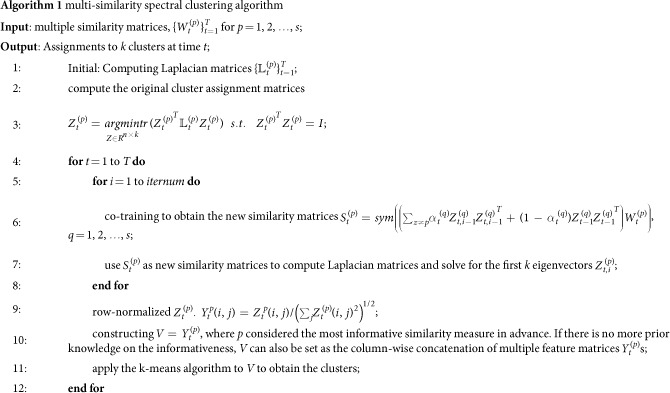


### Determining *α*

We have presented our proposed MSSC method. However, the temporal smoothing parameter 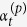 remains unknown, which prevents the clustering result at any snapshot from significantly deviating from the clustering result in the neighbouring snapshot. In many cases, the parameter depends on the subjective preference of the user. To work around this problem, Kevin S. Xu^49^ presented a framework that adaptively estimated the optimal smoothing parameter using shrinkage estimation. In this section, we propose a different approach to adaptively estimate the parameter, which can be defined as


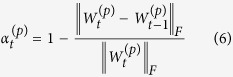


Note that 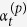 can be easily estimated because 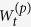 is known. In this model, more weight is placed on the current similarity, because the data should not dramatically shift to the neighbouring snapshot. Further more, a large difference in *W* indicates a small *α*, so it takes more information from the past.

### Changing community numbers

We have assumed that the number of community *k* is fixed, which is a notably strong restriction to the application of our approach. In fact, our approach can handle variations in community numbers. When the community numbers are different at two neighbouring snapshots, the approximation in Equation (5) is free from the effect of changes in clusters, i.e., 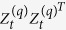 and 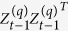 is independent of the community numbers.

### Inserting and removing nodes

In many real-world networks, new nodes join or existing nodes leave the networks often. Assume that at time t, old nodes are removed from and new nodes are inserted into the network. We handle this problem by applying some heuristic solution to transform 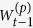 and 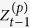 to the same dimension as 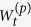 and 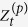, respectively^26^. When old nodes are removed, we can remove the corresponding rows from 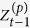 in Equation (5) to obtain 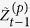
(assuming that 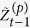 is *n*_1_ × *k*). When new nodes are inserted, we must extend 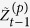 to 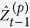, which has the identical dimension as 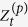
(assuming the dimension of 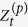 is *n*_2_ × *k*). Then, 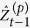 is defined as


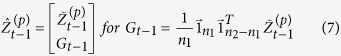


For Equation (6), when old nodes are removed, we can remove the corresponding rows and columns from 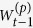 to obtain 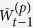 (assuming that 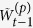 is *n*_1_ × *n*_1_). When new nodes are inserted, we add the corresponding rows and columns to obtain 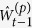, which has the identical dimension as 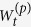
(assuming that the dimension of 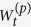 is *n*_2_ × *n*_2_). 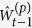 can be defined as


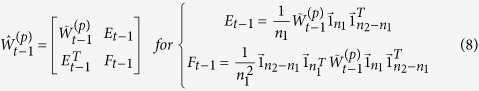


## Additional Information

**How to cite this article**: Qin, X. *et al*. A multi-similarity spectral clustering method for community detection in dynamic networks. *Sci. Rep.* **6**, 31454; doi: 10.1038/srep31454 (2016).

## Figures and Tables

**Table 1:** The performance in different GN-benchmark networks.

	Cn		aveage degree = 16			aveage degree = 20		
			z = 4	z = 5	z = 6	z = 4	z = 5	z = 6
PCQ-NA	1	NMISSE	0.3562 ± 0.04554224.38 ± 260.79	0.0393 ± 0.01786069.06 ± 81.79	0.0253 ± 0.00726101.72 ± 70.14	0.9111 ± 0.0311813.00 ± 340.81	0.5383 ± 0.10743071.78 ± 827.96	0.1129 ± 0.06295636.56 ± 336.75
	3	NMISSE	0.3333 ± 0.04374378.28 ± 289.09	0.0384 ± 0.01696076.68 ± 75.84	0.0252 ± 0.00766131.48 ± 84.28	0.9142 ± 0.0384675.24 ± 263.41	0.5268 ± 0.10423133.74 ± 792.06	0.1055 ± 0.05525682.42 ± 298.19
	6	NMISSE	0.3165 ± 0.05974470.96 ± 366.32	0.0400 ± 0.01386074.34 ± 86.10	0.0253 ± 0.00566119.46 ± 81.34	0.9256 ± 0.0389556.82 ± 342.32	0.5084 ± 0.10723288.68 ± 766.29	0.1059 ± 0.05775686.82 ± 291.96
PCQ-NC	1	NMISSE	0.4059 ± 0.14433815.34 ± 995.63	0.0404 ± 0.02046001.10 ± 107.43	0.0274 ± 0.01056078.12 ± 91.28	0.9034 ± 0.0307898.88 ± 298.82	0.5589 ± 0.11282827.82 ± 838.13	0.1106 ± 0.04765615.80 ± 250.09
	3	NMISSE	0.3921 ± 0.14053898.54 ± 967.97	0.0394 ± 0.01945999.10 ± 106.08	0.0290 ± 0.00996053.18 ± 88.14	0.9288 ± 0.0346503.94 ± 211.46	0.5349 ± 0.12623002.60 ± 943.07	0.1031 ± 0.04285672.86 ± 240.05
	6	NMISSE	0.3670 ± 0.12184021.20 ± 858.63	0.0394 ± 0.01776010.98 ± 88.34	0.0267 ± 0.00986071.34 ± 70.74	0.9137 ± 0.0316646.28 ± 219.35	0.4958 ± 0.10843295.12 ± 783.13	0.0966 ± 0.04245710.98 ± 210.87
PCM-NA	1	NMISSE	0.3116 ± 0.06214539.50 ± 394.25	0.0412 ± 0.01586053.88 ± 104.21	0.0257 ± 0.00566121.96 ± 71.94	0.8999 ± 0.0387810.52 ± 390.29	0.4974 ± 0.11333358.52 ± 784.04	0.1054 ± 0.05685680.16 ± 305.19
	3	NMISSE	0.3109 ± 0.07094545.74 ± 419.80	0.0395 ± 0.01646057.90 ± 93.30	0.0251 ± 0.00726126.38 ± 83.91	0.8877 ± 0.0344948.72 ± 312.82	0.4980 ± 0.11693346.96 ± 797.57	0.1039 ± 0.05425682.32 ± 278.17
	6	NMISSE	0.3098 ± 0.06564550.38 ± 380.21	0.0403 ± 0.01446073.36 ± 81.51	0.0239 ± 0.00526126.62 ± 70.79	0.9294 ± 0.0323467.30 ± 173.87	0.4945 ± 0.11493386.82 ± 824.11	0.1058 ± 0.05595683.04 ± 293.82
PCM-NC	1	NMISSE	**0.6412** ± 0.1278**2155.12** ± 935.50	0.0392 ± 0.01916012.28 ± 114.99	0.0314 ± 0.01256049.72 ± 83.70	0.9004 ± 0.0484889.90 ± 396.37	**0.7819** ± 0.1129**1286.42** ± 812.42	0.1197 ± 0.03705578.26 ± 257.97
	3	NMISSE	**0.4307** ± 0.0931**3550.10** ± 735.88	0.0408 ± 0.01975984.40 ± 107.92	0.0267 ± 0.00576046.64 ± 40.66	0.8737 ± 0.0461860.80 ± 290.84	**0.5893** ± 0.0993**2407.00** ± 657.48	0.0852 ± 0.02425789.26 ± 122.49
	6	NMISSE	0.2804 ± 0.05254627.50 ± 341.80	0.0402 ± 0.01886007.58 ± 77.13	0.0233 ± 0.00676080.16 ± 38.87	0.8951 ± 0.0400701.52 ± 251.36	0.3785 ± 0.09314026.64 ± 570.82	0.0609 ± 0.02395915.96 ± 158.57
StaticSpectral	1	NMISSE	0.3741 ± 0.13153971.22 ± 924.64	0.0396 ± 0.01706012.54 ± 98.31	0.0284 ± 0.01076068.14 ± 108.58	0.9166 ± 0.0289619.68 ± 274.70	0.4940 ± 0.11983305.72 ± 880.41	0.0992 ± 0.04565667.04 ± 268.22
	3	NMISSE	0.3732 ± 0.13163988.72 ± 933.37	0.0394 ± 0.01766005.98 ± 88.83	0.0263 ± 0.01046083.30 ± 106.78	0.9117 ± 0.0349674.82 ± 338.08	0.5000 ± 0.11593260.94 ± 822.32	0.0985 ± 0.04545688.84 ± 235.99
	6	NMISSE	**0.3771** ± 0.1300**3964.94** ± 928.45	0.0421 ± 0.01865997.50 ± 96.17	0.0264 ± 0.01126086.88 ± 106.90	0.9080 ± 0.0483704.44 ± 421.43	0.4898 ± 0.11563351.84 ± 848.26	0.0995 ± 0.04515687.16 ± 233.97
MSSC	1	NMISSE	0.4684 ± 0.05973461.16 ± 471.45	**0.0623** ± 0.0248**5915.66** ± 132.83	**0.0396** ± 0.0094**5996.36** ± 57.92	**0.9806** ± 0.0144**100.40** ± 78.85	0.6462 ± 0.13112284.44 ± 969.86	**0.1352** ± 0.0669**5484.36** ± 363.86
	3	NMISSE	0.4108 ± 0.07473840.66 ± 526.34	**0.0562** ± 0.0200**5957.56** ± 86.20	**0.0378** ± 0.0099**6018.72** ± 80.61	**0.9693** ± 0.0238**154.56** ± 120.58	0.5639 ± 0.11422836.34 ± 844.02	**0.1257** ± 0.0571**5560.14** ± 319.88
	6	NMISSE	0.3727 ± 0.07024059.82 ± 471.45	**0.0551** ± **0**.**01835959.74** ± 113.17	**0.0405** ± 0.0089**6013.50** ± 81.21	**0.9641** ± 0.0276**182.72** ± 140.66	**0.5428** ± 0.1113**3063.80** ± 831.10	**0.1265** ± 0.0594**5548.00** ± 336.35

When parameter z = 4, 5 and 6, the average degree of each node is 16 and 20 at each snapshot, we randomly select 1, 3 and 6 nodes change their cluster membership, respectively. Notice that the value of NMI and SSE is the average for 10 snapshots.

**Table 2:** The performance in different GN-benchmark networks #2.

	z	syn-fix		syn-var	
		NMI	SSE	NMI	SSE
PCQ-NA	35	0.6028 ± 0.20330.6069 ± 0.2016	9640.60 ± 4779.749340.80 ± 4642.21	0.6132 ± 0.18620.6116 ± 0.1928	9259.42 ± 3910.069174.40 ± 4197.49
PCQ-NC	35	**0**.**6133** ± 0.18400.6054 ± 0.1884	9319.38 ± 4134.379466.48 ± 4214.53	0.6002 ± 0.17980.5984 ± 0.1922	9841.54 ± 3830.969656.26 ± 4299.85
PCM-NA	35	0.5963 ± 0.19960.5993 ± 0.1978	9460.58 ± 4666.489304.36 ± 4568.22	0.5926 ± 0.18720.5834 ± 0.1928	9720.78 ± 3991.919855.60 ± 4235.54
PCM-NC	35	0.5978 ± 0.1862**0**.**6109** ± 0.1943	9916.08 ± 4193.829271.82 ± 4401.95	0.6070 ± 0.18540.6139 ± 0.1907	9536.20 ± 3861.539123.94 ± 4136.11
StaticSpectral	35	0.5835 ± 0.19540.5781 ± 0.2052	9668.48 ± 4259.439755.16 ± 4679.10	0.5863 ± 0.18960.5812 ± 0.1862	9482.16 ± 3893.939659.56 ± 3723.53
MSSC	35	0.5852 ± 0.20520.6091 ± 0.2094	**2340**.**58** ± 1232.92**2171**.**14** ± 1246.76	**0**.**6458** ± 0.1805**0**.**6475** ± 0.1747	**8261**.**06** ± 4294.08**8263**.**74** ± 4162.19

The performance for SYN-FIX and SYN-VAR with *z* = 3 and *z* = 5, respectively. For SYN-FIX, the number of communities is fixed. For SYN-VAR, a new community is created once at each timestamp between 2 ≤ *t* ≤ 5.

**Table 3:** The performance for five dynamic networks.

		birthdeath	expand	contraction	mergesplit	switch
PCQ-NA	NMISSE	0.8398 ± 0.011874170.32 ± 18518.48	0.8485 ± 0.012290065.30 ± 13012.11	0.8365 ± 0.017594808.78 ± 19132.44	0.8515 ± 0.009684288.78 ± 8577.71	0.8381 ± 0.0187101091.10 ± 20912.82
PCQ-NC	NMISSE	0.8504 ± 0.024768511.50 ± 20307.64	0.8457 ± 0.017692582.98 ± 14792.11	0.8430 ± 0.014389217.64 ± 15869.03	0.8373 ± 0.016094506.46 ± 15785.94	0.8432 ± 0.014397056.32 ± 16190.39
PCM-NA	NMISSE	0.8356 ± 0.012977350.20 ± 21198.80	0.8368 ± 0.017599648.56 ± 16180.79	0.8316 ± 0.017597976.98 ± 17861.72	0.8374 ± 0.018890110.12 ± 14109.52	0.8446 ± 0.011793093.64 ± 13556.13
PCM-NC	NMISSE	0.8419 ± 0.016874547.64 ± 16656.38	0.8369 ± 0.0193101543.20 ± 17771.21	0.8469 ± 0.017283593.96 ± 18388.87	0.8407 ± 0.019090853.56 ± 12056.83	0.8359 ± 0.0153101398.38 ± 15773.68
StaticSpectral	NMISSE	0.8548 ± 0.017652299.48 ± 12337.69	0.8574 ± 0.015470764.06 ± 8715.67	0.8540 ± 0.021970587.36 ± 12970.81	0.8477 ± 0.016075613.28 ± 9825.15	0.8480 ± 0.0193101398.38 ± 12010.70
MSSC	NMISSE	**0**.**9335** ± 0.0076**18842**.**36** ± 5317.05	**0**.**9303** ± 0.0046**26923**.**20** ± 2996.34	**0**.**9284** ± 0.0076**27066**.**20** ± 5976.27	**0**.**9306** ± 0.0082**25206**.**58** ± 3995.91	**0**.**9360** ± 0.0084**24462**.**02** ± 4547.29

Dynamic networks for five different event type: birth and death, expansion, contraction, merging and splitting, switch nodes.

**Table 4:** The performance for the KIT E-mail Dataset.

		T = 48	T = 24	T = 16	T = 12	T = 8
PCQ-NA	NMISSE	0.7567 ± 0.0365369.70 ± 58.83	0.7850 ± 0.03981259.69 ± 265.12	0.7760 ± 0.03961915.06 ± 463.29	0.7479 ± 0.03602766.32 ± 581.48	0.7362 ± 0.04163892.13 ± 1007.15
PCQ-NC	NMISSE	0.8120 ± 0.0401253.33 ± 60.63	0.8105 ± 0.0284924.83 ± 156.76	0.7933 ± 0.02871371.71 ± 202.50	0.7807 ± 0.02921862.48 ± 215.96	0.7736 ± 0.02152476.00 ± 224.26
PCM-NA	NMISSE	0.7466 ± 0.0433382.39 ± 69.97	0.7773 ± 0.03861290.58 ± 258.89	0.7680 ± 0.04341949.60 ± 462.78	0.7378 ± 0.03982856.55 ± 628.81	0.7326 ± 0.04003954.75 ± 1018.30
PCM-NC	NMISSE	0.8290 ± 0.0345**232**.**27** ± 58.91	0.8150 ± 0.0214924.99 ± 109.74	0.8076 ± 0.02481296.94 ± 174.63	0.7796 ± 0.03171884.85 ± 230.36	0.7680 ± 0.02032686.10 ± 152.40
StaticSpectral	NMISSE	0.8018 ± 0.0398265.45 ± 56.69	0.8059 ± 0.0249933.73 ± 135.10	0.7871 ± 0.02841418.89 ± 226.12	0.7795 ± 0.02871848.40 ± 201.88	0.7674 ± 0.02122518.20 ± 224.53
MSSC	NMISSE	**0**.**8333** ± 0.0214241.52 ± 28.73	**0**.**8448** ± 0.0206**846**.**61** ± 77.28	**0**.**8271** ± 0.0273**1297**.**94** ± 177.73	**0**.**8086** ± 0.0270**1676**.**28** ± 226.60	**0**.**8021** ± 0.0196**2328**.**38** ± 177.97

The e-mail networks taking 1, 2, 3, 4, 6 months as a snapshot, respectively.
